# Assessment of the Effectiveness of Hip Protector Pads Produced by Treated Warp Knitted Spacer Fabrics

**DOI:** 10.1021/acsomega.5c05406

**Published:** 2025-07-23

**Authors:** Gözde Ertekin

**Affiliations:** Faculty of Engineering, Department of Textile Engineering, 37509 Ege University, 35040 İzmir, Turkey

## Abstract

The objective of this study is to assess the effectiveness of hip protector pads utilizing warp knitted spacer fabrics treated with silicone and latex, specifically designed for hip protective applications. Three different types of warp knitted spacer fabrics and silicone- or latex-treated variants were evaluated in terms of transmitted force, compression resistance, and thermo-physiological comfort parameters. A drop weight impact tester with a real hip impact area was used to measure the force attenuation capacity of hip protective pads, yielding transmitted force values ranging from 14.39 to 31.83 kN under applied energies of 12.5 and 25.0 J. Compression resistance values varied from 219 to above 950 N, depending on the fabric structure and treatment. Air permeability of the fabrics ranged from 1300 to 6000 l/m^2^s, thermal resistance values from 0.0355 to 0.0714 m^2^K/W, and water vapor resistance values from 2.66 to 3.99 m^2^ Pa/W. The findings demonstrate that treated warp knitted spacer fabrics—especially both sides open-structured ones—showed higher force attenuation, moderate compression resistance, and improved breathability, making them suitable candidates for hip protective pads that combine protection with prolonged wear comfort.

## Graphical abstract

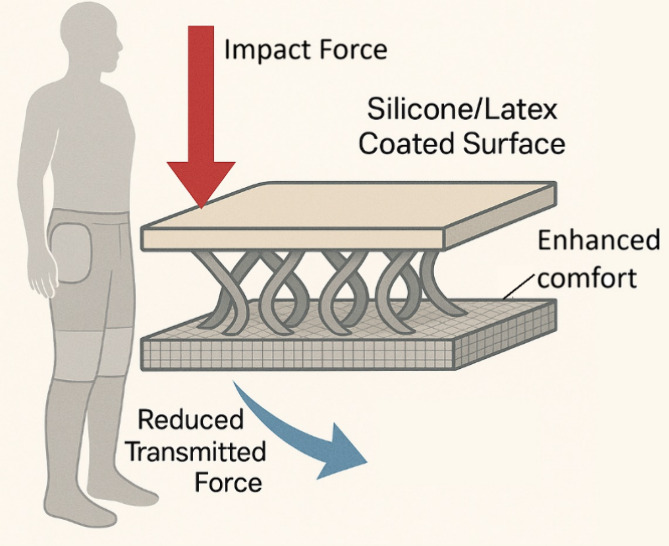

## 1 Introduction

The global elderly population is increasing rapidly, surpassing other age groups in terms of demographic expansion. This trend in aging demographics is anticipated to lead to an increased incidence of disability and various health-related issues. Among individuals aged 65 years and above, falls are identified as the primary cause of injuries. It is projected that by the year 2050, the occurrence of hip fractures will notably escalate due to the increase in the aging population of the world .^1^

The leading cause of hip fractures is a sideway fall, which results in a high-energy impact and increases the chance of a hip fracture by six times compared to other types of falls.^2–4^ The prevalence of hip fractures in the population indicates that approximately 25% of women and 12.5% of men will encounter such injuries during their lifetime.^5^

For healthy aging, reducing hip fractures induced by sideway falls has become more crucial. For the prevention of fractures, a few approaches have been developed, including exercise, calcium and vitamin D supplementation, specialized medications to prevent or cure osteoporosis, and comprehensive interventions to reduce the risk of falling.^6^ On the other hand, a sideway fall with a direct impact on the greater trochanter of the proximal femur is the primary cause of a hip fracture.^7^ Therefore, a logical way to protect the hips and reduce the risk of fractures is to wear hip protective clothing.

A special underwear with integrated hard or soft shields that are worn on the hip to cover the greater trochanter is known as a hip protective garment. It is intended to partially avoid hip fractures and injuries by transferring the kinetic impact energy produced by a sideway fall to soft tissues and muscle.^8,9^ Hip protectors and other passive preventative measures are more successful at preventing hip fractures than strategies designed to delay bone loss in the elderly.^10^ The effectiveness of hip protectors is determined by two factors, mechanical characteristics and wearing time, which are influenced by user compliance and adherence. Adherence is a main problem in hip protective clothing. Hip protective garment compliance may be adversely affected by some variables, including discomfort during use, being overly tight, requiring assistance in toileting,^11,12^ and being difficult to put on.^13^ The type of pad is crucial in addressing these drawbacks. In order to enhance wearers’ compliance, the disadvantages of hip protectors should be addressed, but it should be taken into consideration that, above all other characteristics, the primary criterion for a hip protective pad is its protection performance.

In recent years, several studies have extensively focused on the performance and comfort characteristics of hip protector pads, which are produced using a variety of structures, materials, and treatments.^9,14–23^ For instance, while some advanced designs, including those utilizing additive manufacturing and novel materials such as shear thickening fluid (STF)-filled warp knitted spacer fabrics, demonstrate significant protective capabilities—such as over 82% impact reduction and effectively maintaining impact forces below the 3.47 kN fracture threshold for an elderly woman’s hip bone during a fall,^15,16^ with other commercial pads offering 18.7–46.5% attenuation^16^ —user acceptance and adherence remain critical challenges, underscoring the need for continued research.^17,18^ Compliance with conventional garment-based hip protectors often averages less than 50%, with adherence reported to decline to under 25% in acute care settings,^18^ and over two-thirds of initial wearers stopping within 3 months in some studies.^19^ This low adherence is primarily attributed to persistent comfort and usability issues,^9,14,18–22^ including perceptions of garments being ‘too hot’,^14,18,19,21,22^ too bulky,^17,21,22^ poorly fitting,^9,14,19–21^ or difficult to manage for daily activities and toileting^14,18,20^ with 34% of women in one study refusing them due to discomfort alone.^21^ Therefore, despite significant protective advancements, continued investigation into new materials and designs that balance high impact attenuation with improved real-world wear comfort and practical acceptance is crucial to enhance their effectiveness in preventing hip fractures.^16,20,21,23^

This paper focused on the evaluation of the effectiveness of the treated warp knitted spacer fabrics designed for hip protectors in terms of force attenuation and physical, mechanical, and thermal comfort properties. The protective (transmitted force values), physical (mass per unit area and thickness values), and lifetime properties (compression resistance, dimensional stability, and compression set values) and thermal comfort characteristics (air permeability, thermal resistance, and water vapor resistance) were determined. The effects of surface structure and type of treatment on the above-mentioned properties were investigated. Additionally, a mechanical testing device was developed by placing a real impact area (an upper leg profile and artificial flesh) on the drop weight impact tester for the determination of the hip protectors’ effectiveness.

## 2 Materials and Methods

### 2.1 Materials

A hip protector pad was fabricated using a warp knitted spacer fabric composed of 100% polyester yarns. In the process of fabric production, the spacing between the needle bars was set at 12.5 mm. The experimental design involved the utilization of three different surface structure combinations. The first group consists of a closed structure on both sides of the fabric, the second group has a net-like open structure created with interconnected wales, and the third group consists of a net-like open structure on the face and a closed structure on the back side of the fabric (Figure 1). The yarn count for the face and back structures was 334 dtex, and the spacer yarn was a monofilament of 680 dtex (Table 1).

**Figure 1:**
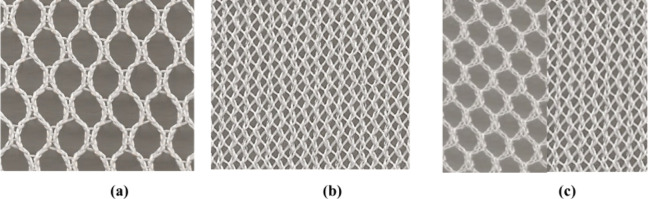
Surface views of (a) a net-like open structure on both sides of the fabric, (b) a closed structure on both sides of the fabric, and (c) a net-like open structure on the face and a closed structure on the back side of the fabric.

**Table 1:** Specifications of the Fabric Samples^a^

no	code	description of material	treatment	mass per unit area (g/m^2^)	thickness (mm)
1	S/C	polyester knitted spacer fabric/closed structure on both sides of fabrics		930.69	10.49
2	S/O	polyester knitted spacer fabric/open structure on both sides of fabrics		683.57	10.80
3	S/OC	polyester knitted spacer fabric/open structure on the face and closed structure on the back side of fabrics		514.45	9.84
4	SST/C	treated polyester knitted spacer fabric/closed structure on both sides of fabrics	silicone coating	1952.00	10.40
5	SST/O	treated polyester knitted spacer fabric/open structure on both sides of fabrics	silicone coating	1402.67	10.70
6	SST/OC	treated polyester knitted spacer fabric/open structure on the face and closed structure on the back side of fabrics	silicone coating	1072.00	9.39
7	SLT/C	treated polyester knitted spacer fabric/closed structure on both sides of fabrics	latex coating	1253.33	11.00
8	SLT/O	treated polyester knitted spacer fabric/open structure on both sides of fabrics	latex coating	922.67	11.49
9	SLT/OC	treated polyester knitted spacer fabric/open structure on the face and closed structure on the back side of fabrics	latex coating	730.67	10.54

a S, spacer fabric—neat; SST, spacer fabric—silicone-treated; SLT, spacer fabric—latex-treated; O, open structure; C, closed structure; OC, one side open, one side closed structure.

### 2.2 Treatment

The warp knitted spacer fabrics were subjected to treatment with either silicone or latex materials using the vacuum infusion technique, with the aim of enhancing the force attenuation capacity of the fabrics. A silicone substrate with two components (10:1 ratio) obtained from ACC Silicons Company was thoroughly mixed using a laboratory mixer. Prior to application, the mixture was subjected to vacuum treatment to eliminate any air bubbles (Table 2).^24^ The fabrics were coated with this substrate in a 1:1 weight ratio. The latex utilized in this study was a synthetic latex obtained through the emulsion polymerization of styrene and butadiene, resulting in a copolymer containing a substantial amount of styrene. The fabrics were coated with this substrate in a 1:1/2 weight ratio.

**Table 2:** Properties of the Commercial Silicone Rubber

viscosity (mPa s)	tensile strength (MPa)	elongation at break (%)	Young’s Modulus (MPa)	tear strength (kN m^–1^)	hardness
96.000	5.40	330.00	1.88	22.00	40° Shore A

The vacuum infusion process was used for the coating of fabric samples. The warp knitted spacer fabrics were placed on the surface of the mold. A sealant tape was applied to the vacuum bag to adhere to the mold. The silicone and latex substrates was infused under vacuum using a vacuum pump at pressure up to 1 bar (Figure 2). Following the complete infusion of fabric samples with the necessary resin, they were subjected to a 24 h curing process at 25 °C. The coated fabric samples were postcured in an oven at a temperature of 80 °C for 4 h.^25^ Images of samples of the treated warp knitted spacer fabrics and of an undergarment with pockets that contain silicone- or latex-coated warp knitted spacer fabrics are displayed in Figures 3 and 4, respectively.

**Figure 2:**
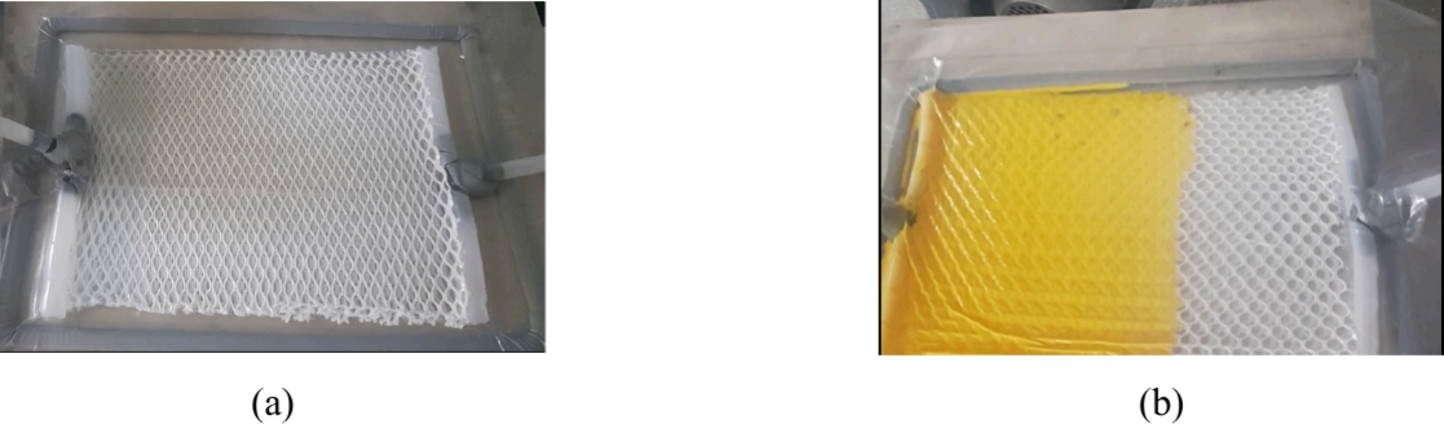
Fabric samples coated with (a) silicone and (b) latex during the vacuum infusion process.

**Figure 3:**
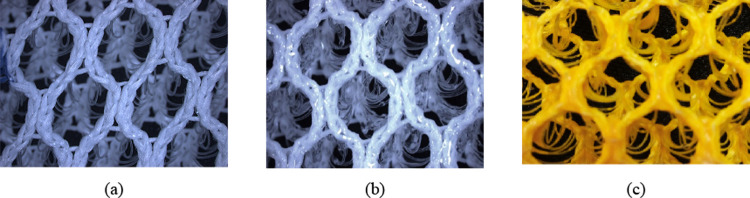
Surface views of (a) neat, (b) silicone-coated, and (c) latex-coated warp knitted spacer fabrics.

**Figure 4:**
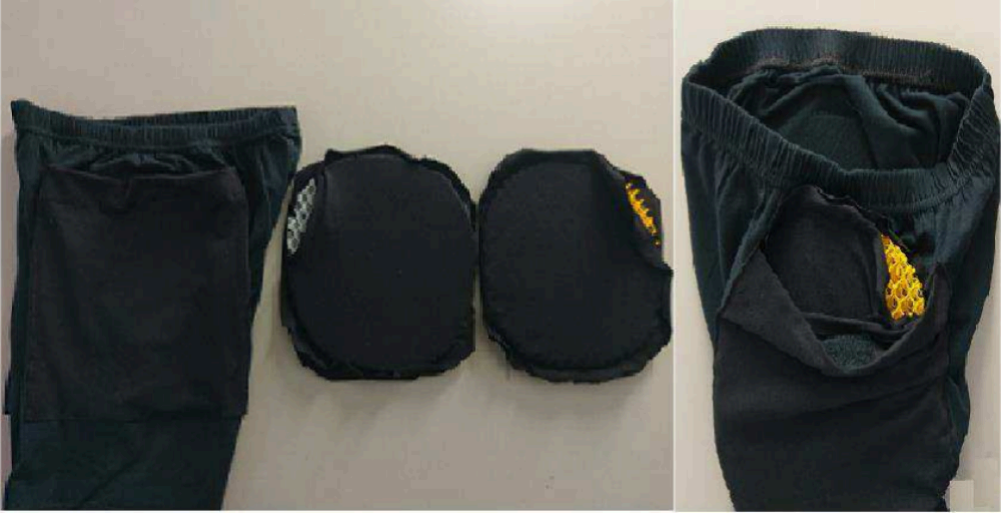
Images of a sample of an undergarment with pockets that contain silicone- or latex-coated warp knitted spacer fabrics.

### 2.3 Methods and Statistical Analysis

The physical properties such as fabric thickness (measured with a caliper) and mass per unit area (according to TS EN 12127) of the spacer fabric samples before and after the coating process were measured and are shown in Table 1.

For the determination of the force attenuation capacity of the spacer fabrics, transmitted force values were measured using a drop weight impact tester according to the standard BS EN 1621-1 (Motorcyclist’s protective clothing against mechanical impact—Part 1: Requirements and test methods for impact protectors), as shown in Figure 5a. To assess the efficacy of the hip protectors, a realistic impact area representing the upper leg profile of an adult weighing 65 kg and measuring 1.75 m in height was fabricated. This impact area was positioned on a load cell (Figure 5b), which measured the transmitted force value in place of a conventional anvil. The desired impact energy was obtained by changing the falling height of the striker. A tissue-like structure is used as the outer part material to simulate the soft tissue, and the upper leg profile is covered with this material.^26^ It has a density of 1.2 kg m^–3^ and shore A hardness of 12, the same properties as the artificial flesh used in previous studies.^17,27^ The impact energy of the dropper was adjusted by changing the falling height. According to Robinovitch et al.,^28^ typical speeds of a person's fall are 2.0–2.5 and 3.0–3.4 ms^–1^ for a mild and moderate fall, respectively. Equation 1 illustrates the kinematic equation of a free fall.

$$V^{2}=2gh$$ 1

where *V* is the speed (ms^–1^), *g* is the gravity (ms^–2^), and *h* is the height (m).

**Figure 5:**
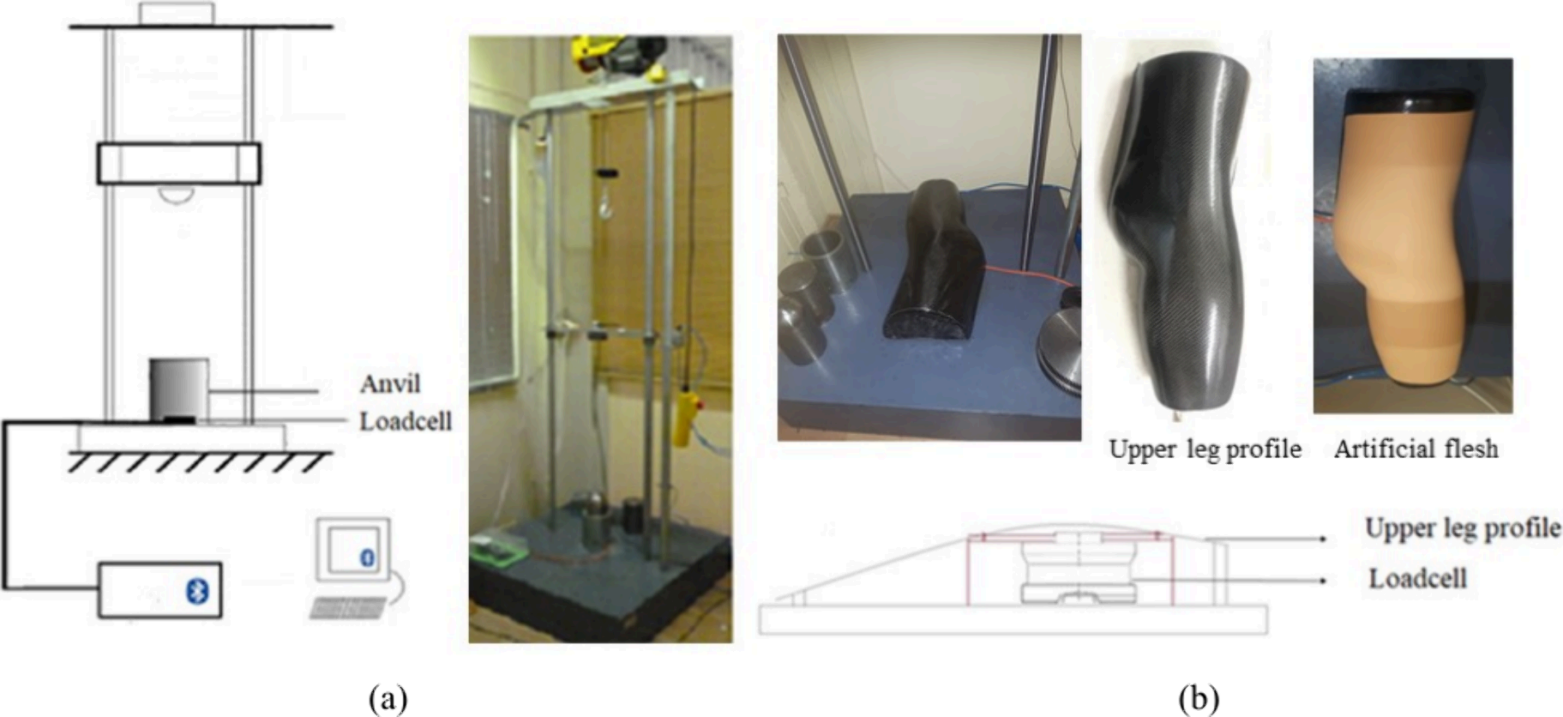
(a) Drop weight impact tester and (b) upper leg profile placed on the load cell.

The falling height can be calculated by rearranging the figure (eq 1) as follows.

$$h=\frac{V^{2}}{2g}$$ 2

The falling heights according to mild and moderate speeds of 2.5 and 3.0 ms^–1^ are 2.5 and 0.5 m, respectively. The weight of the dropper is (5000 ± 10) g. The impact energy of the falling object is

E=mgh 3

where *E* is the impact energy (J), *m* is the mass of the dropper (kg), and *h* is the falling height (m).

According to eq 3, the impact energies were calculated as 12.5 and 25.0 J. These energy values were used for the determination of the effect of impact energy levels on the fabric parameters. The peak transmitted force was recorded, and the data were transferred to a computer using Bluetooth. Five specimens were tested for each fabric.

The performance characteristics of the spacer fabrics such as compression resistance, dimensional stability, and compression set were determined. Compression resistance tests were carried out by a Zwick R010 Instrument based on the ISO 3386 standard. 950N was applied to the samples, and the set compression rate is 25%. The measurement was performed by using two layers of fabric samples in both the course and wales directions. The results are averages of three readings in N. The dimensional stability was tested according to the DIN 53377 standard. The samples are cut into a square form of 10 × 10 cm and are placed in an oven maintained at 90 °C for 1 h. The dimensional stability value was calculated according to eq 4:

$$\dim\mathrm{ensional}\;\mathrm{stability}\;(\%)=\frac{\left(l_{0}-l_{F}\right)}{l_{0}}\times 100$$ 4

where *l*_0_ represents the original dimensions of the specimen, and *l*_F_ represents the dimensions of the specimen after treatment.

The permanent deformation is the difference between the initial thickness and the final thickness of a test piece of the material after compression for a given time at a given temperature and after a given recovery time and is determined according to the TS 2013 EN ISO 1856 standard. The samples (50 × 50 × 25 mm) are compressed at a rate of 75% and are placed in an oven maintained at 70 °C for 22 h. The permanent deformation value was calculated according to eq 5:

$$\mathrm{Permanentde}\;\mathrm{formation}(\%)=\frac{\left(d_{0}-d_{r}\right)}{d_{0}}\times 100$$ 5

where *d*_0_ is the original thickness of the specimen, and *d*_r_ is the thickness value of the specimen waited for 12 h after experiment.

The silicone and latex coatings were characterized to assess their chemical structures and surface morphologies. Fourier transform infrared (FTIR) spectroscopy was performed using a PerkinElmer spectrophotometer in the range of 4000–800 cm^–1^. The surface morphology was evaluated by scanning electron microscopy (SEM) using a Philips XL-30S FEG scanning electron microscope.

The thermal comfort properties such as air permeability, thermal resistance, and water vapor resistance properties were measured using a Textest FX 3300 instrument and Permetest according to the TS 391 EN ISO 9237 and ISO EN 11092 standards, respectively. The results of the measurements are averages from the values of 10 readings for air permeability and three readings for thermal resistance and water vapor resistance.

The data obtained from the performance, comfort, and impact resistance characteristics were evaluated using one-way analysis of variance (ANOVA). Any differences for each dependent variable were considered significant if the *p*-value was equal to or less than 0.05.

## 3 Results and Discussion

### 3.1 Effect of Treatment Types and Fabric Surface Structures

All measured values are presented as the mean ± standard deviation (SD) in Table 3. Error bars in Figures 6–12 represent the standard deviation of repeated measurements (*n* = 5 for impact tests, *n* = 10 for air permeability tests, and *n* = 3 for other investigated parameters). Also, the *p* values of the investigated parameters are given in Table 4. According to the statistical evaluation, the structure of fabric surfaces and the type of treatments had significant effect on all investigated parameters.

**Table 3:** Mean ± Standard Deviation Values of the Investigated Parameters

			compression resistance (N)		dimensional stability (%)	
code	weight (g/m^2^)	thickness (mm)	course-wise	Wales-wise	course-wise	Wales-wise
S/C	930.69 ± 18.72	10.49 ± 0.04	307 ± 1.63	300 ± 1.63	–1	0
S/O	683.57 ± 9.42	10.80 ± 0.09	280 ± 1.63	219 ± 1.62	–2	0
S/OC	514.45 ± 10.56	9.84 ± 0.13	272 ± 1.64	250 ± 1.67	–3	0
SST/C	1952.00 ± 22.63	10.40 ± 0.14	950 ± 0.00	950 ± 0.00	0	0
SST/O	1402.67 ± 19.96	10.70 ± 0.13	627 ± 1.64	539 ± 1.61	–1	0
SST/OC	1072.00 ± 91.45	9.39 ± 0.10	440 ± 1.62	421 ± 1.65	–1	0
SLT/C	1253.33 ± 15.09	11.00 ± 0.11	950 ± 0.00	950 ± 0.00	0	0
SLT/O	922.67 ± 19.96	11.49 ± 0.17	647 ± 1.63	604 ± 1.63	–1	0
SLT/OC	730.67 ± 7.54	10.54 ± 0.16	465 ± 1.63	443 ± 1.64	–1	0

	transmitted force (kN)		permanant deformation (%)	air permeability (l/m^2^ s)	thermal resistance (m^2^ K/W)	water vapor resistance (m^2^ Pa/W)
code	25.0 J	12.5 J				
S/C	31.33 ± 0.02	20.93 ± 0.50	28.97 ± 0.45	2972.00 ± 195.43	0.0714 ± 0.00	2.95 ± 0.02
S/O	29.76 ± 0.25	20.17 ± 0.02	31.10 ± 0.67	6024.00 ± 102.12	0.0502 ± 0.00	2.66 ± 0.02
S/OC	31.83 ± 0.02	21.19 ± 0.02	33.25 ± 0.78	5142.00 ± 179.53	0.0618 ± 0.00	2.89 ± 0.02
SST/C	27.22 ± 0.61	17.06 ± 0.42	21.02 ± 0.24	1343.80 ± 127.85	0.0573 ± 0.00	3.99 ± 0.02
SST/O	21.46 ± 0.02	14.39 ± 0.29	23.08 ± 0.22	5691.33 ± 185.32	0.0355 ± 0.00	2.79 ± 0.02
SST/OC	31.34 ± 0.15	19.68 ± 0.06	25.28 ± 0.33	4354.00 ± 259.20	0.0581 ± 0.00	3.02 ± 0.02
SLT/C	30.28 ± 0.23	18.14 ± 0.14	19.20 ± 0.37	2316.67 ± 540.79	0.0656 ± 0.00	3.95 ± 0.02
SLT/O	24.64 ± 0.98	14.56 ± 0.55	20.95 ± 0.31	5665.33 ± 178.99	0.0478 ± 0.00	2.75 ± 0.02
SLT/OC	31.66 ± 0.22	19.74 ± 0.02	22.94 ± 0.23	4644.67 ± 203.76	0.0607 ± 0.00	2.98 ± 0.02

**Table 4:** *p* Values of the Investigated Parameters

	*F*	sig.
transmitted force at 25.0 J	51.081	0.000^a^
transmitted force at 12.5 J	8.014	0.000^a^
compression resistance (course-wise)	68328.290	0.000^a^
compression resistance (wales-wise)	76257.550	0.000^a^
permanent deformation	14.656	0.001^a^
air permeability	964.751	0.000^a^
thermal resistance	734.213	0.000^a^
water vapor resistance	1951.690	0.000^a^

a Significant at the 0.05 level.

**Figure 6:**
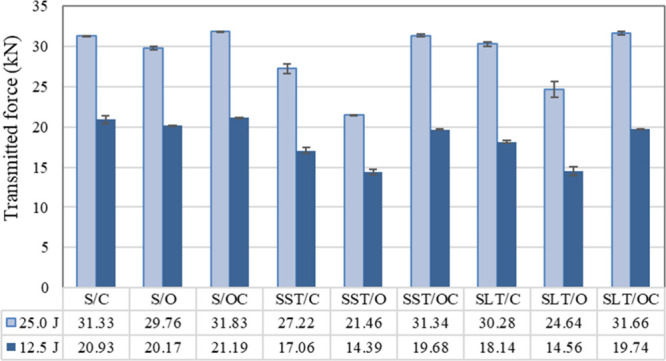
Transmitted force values of the samples under 12.5 and 25.0 J applied impact energies.

Figure 6 presents the transmitted force values of the fabrics under applied energies of 12.5 and 25.0 J. One-way ANOVA test showed that there are significant differences between the transmitted force values of the hip protector pads (*p* < 0.05). After both silicone and latex treatment, the transmitted force values of the fabrics at impact energies of 25.0 and 12.5 J decreased by approximately 4 and 2 kN for closed-structured pads and 8 and 6 kN for open-structured ones, respectively. Especially for both sides closed-structured or both sides open-structured fabrics, silicone coating provides higher impact protection performance than latex coating. The force attenuation capacity of a material can be increased by increasing the exposure time against impact or by distributing the load over a larger area. The thickness of the material has a significant effect on the transmitted force values, and as the thickness increases, the transmitted force decreases, improving the impact resistance properties of the materials. The fabric with a face-side open and back-side closed structured configuration, exhibiting the lowest thickness values, demonstrated inferior protection characteristics, as indicated by the highest transmitted force values. Regarding both treatments, the pads with the both sides open-structured configuration exhibited the highest protection capacity, characterized by higher thickness values.

For both impact energy levels, the silicone- and/or latex-coated pads with the both sides open-structured configuration demonstrated the lowest transmitted force values, making them preferable as protective pads, specifically concerning the impact protection performance. The effectiveness of the experimental pads was compared to two commercially available hip protector brands. Two different brands of hip protectors were provided and assessed using the drop weight impact tester under equivalent impact energy levels. The results revealed that Brand A and Brand B hip protectors had transmitted force values of 26.28 and 25.13 kN at an impact energy level of 25.0 J and 15.62 and 14.98 kN at an impact energy level of 12.5 J, respectively.

While an increase in fabric thickness generally contributes to improved impact attenuation by reducing the transmitted force, it may also negatively affect the thermo-physiological comfort and wearability of the hip protector pads. Excessively thick structures can cause discomfort, restrict movement, and reduce user compliance. Therefore, a balance between thickness and comfort is crucial. In this study, both sides of the open-structured pads treated with silicone or latex exhibited a relatively higher thickness and protection performance, while maintaining moderate compression resistance and favorable air permeability. These findings suggest that this configuration may provide a favorable compromise between protection and comfort, making it suitable for prolonged daily use in wearable applications.

Compression is one of the important fabric properties, in addition to friction, bending, tension, and shear. The internal forces of the fibers and the frictional forces between the fibers must be overcome by the force required to compress a fabric. Given how quickly it compresses, a fabric with a low compression resistance or high compressibility is likely to be considered as soft.^29,30^ The compressibility of three-dimensional textiles is influenced by several factors, including the fabric’s thickness, density, bending characteristics, and the inclination angle of the spacer yarn.

The silicone or latex treatments improved the compression resistance of the pads in both directions. Among the treated pads, those coated with latex exhibited higher compression resistance compared with the ones coated with silicone. The compression resistance values of the treated pads with the both sides closed-structured configuration exceeded the measurement range of the device and thus could not be measured. The compression resistance of both sides of the closed-structured pad was higher due to its more stable and rigid structure. The face-side open and back-side closed structured pad exhibited the lowest resistance against compression due to its lower mass per unit area and thickness values, resulting in a lower fabric density (Figure 7). On the other hand, both sides of open-structured pads treated with silicone and/or latex showed moderate compression resistance among the fabrics studied in this investigation.

**Figure 7:**
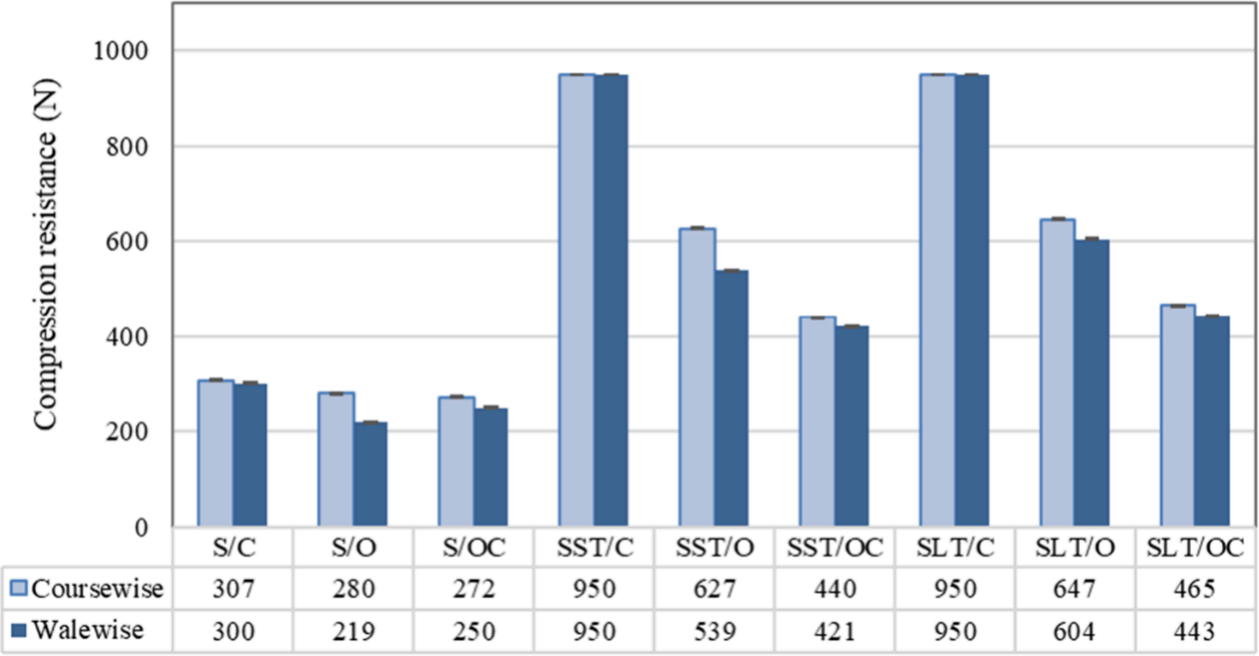
Compression resistance behavior of the samples.

The degree to which a fabric maintains its original length and width is known as its dimensional stability. It is usually preferable for textiles to have a higher dimensional stability. Shrinkage is the term for a reduction in dimensions, whereas growth is the term for an increase in dimension. Fabrics generally shrink during the production and washing because of the relaxation of the fibers/yarns, swelling of the fibers, and felting.^31,32^

The dimensional stability of the pads was tested in the course-wise and widthwise directions (Figure 8). According to the results, no dimensional change in the wales-wise direction was observed in any of the samples. Treatment with either silicone or latex increased the dimensional stability in the course-wise direction for all surface pattern types. The shrinkage values decreased to 1% after treatments. The results revealed that treatment has a dominant effect on the dimensional stability rather than surface pattern types. Fabrics with both sides closed surfaces demonstrated higher dimensional stability compared to the other two types of pads.

**Figure 8:**
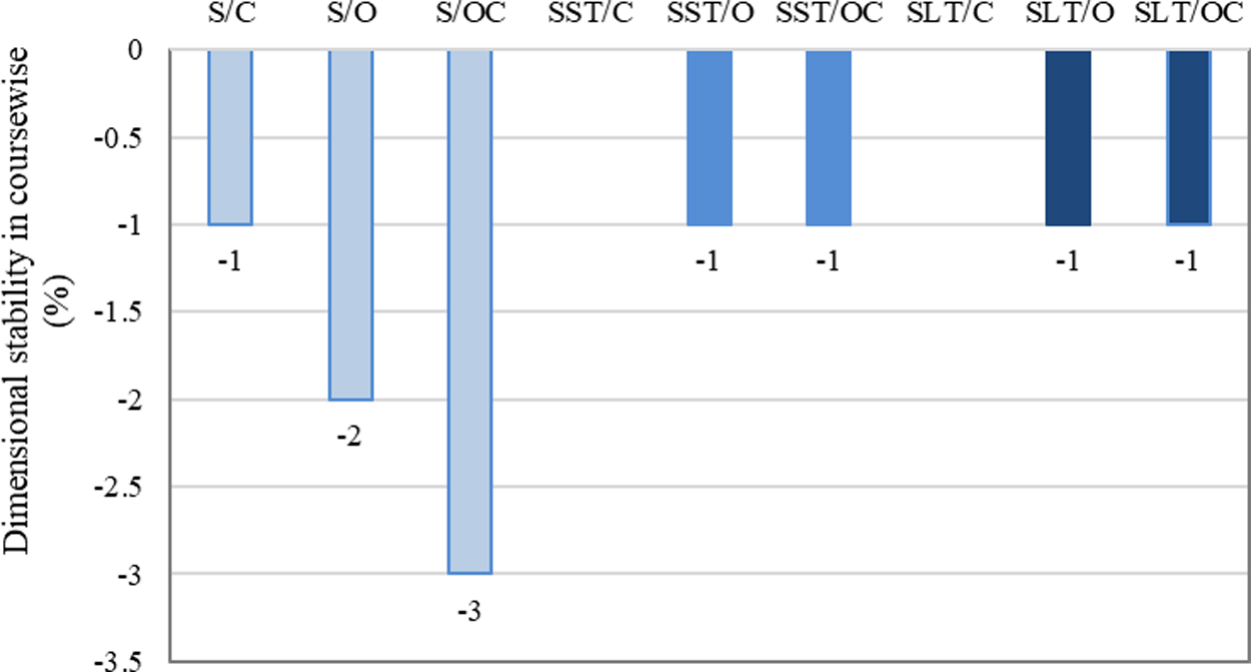
Dimensional stability of the samples.

One of the key variables affecting the durability and lifespan of the materials is permanent deformation, generally known as the aging test. There is an inverse relation between the durability and permanent deformation characteristics of the pads. The lower the permanent deformation, the higher the endurance of the samples.^28^

ANOVA test showed that there is a significant difference between the permanent deformation values of the pads (*p* < 0.05). The results showed that treatment enhanced the durability of the pads, and among all groups, latex-treated pads exhibited a longer lifetime due to their lower permanent deformation values (Figure 9).

**Figure 9:**
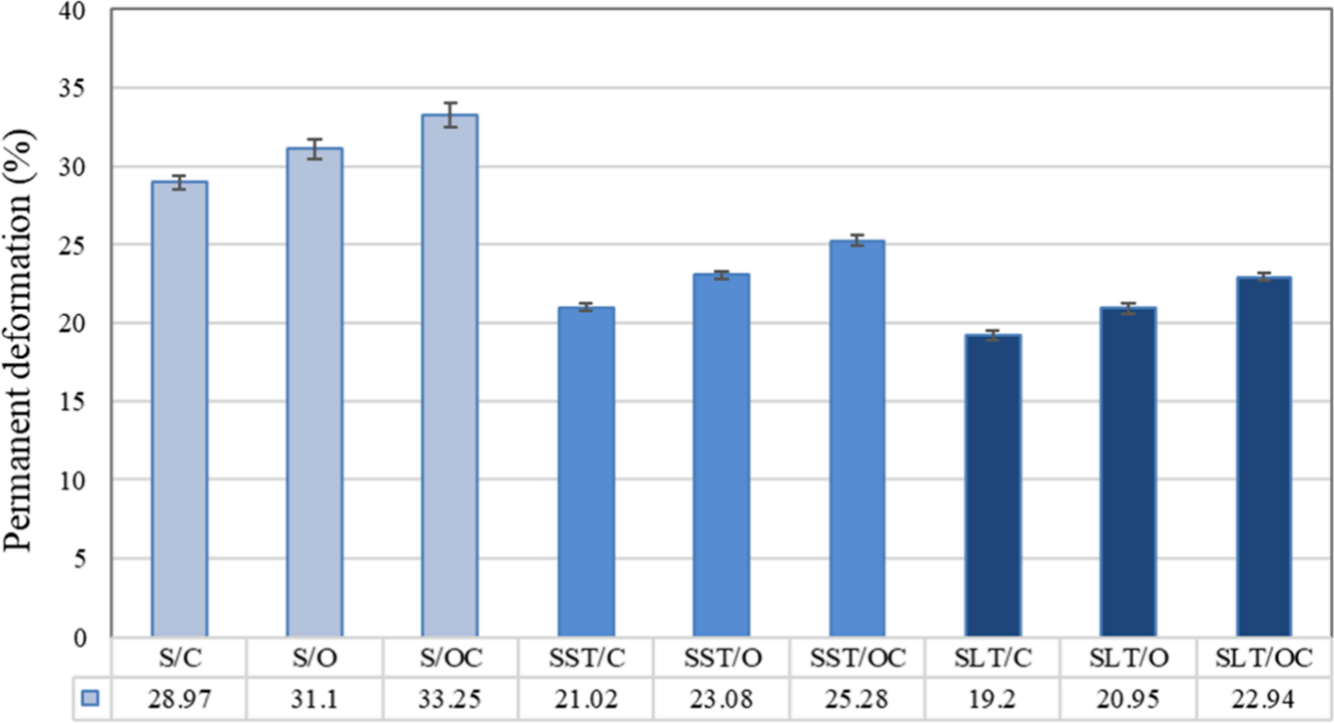
Permanent deformation of the samples.

In the investigation of surface structure influence, it was observed that spacer fabrics with both sides closed structures exhibited the lowest permanent deformation values, indicating greater durability over time. The results indicated that the fabric surface structure, coating, and density collectively influenced its compression resistance capabilities. In the comparative analysis of treatment types, it is observed that latex-coated fabrics exhibit lower permanent deformation values despite possessing a higher fabric density compared to silicone-coated fabrics. Typically, fabrics with a higher fabric density tend to display reduced permanent deformation tendencies. However, the seeming contradiction between the fabric density and permanent deformation in latex-coated fabrics can be elucidated by considering the unique material properties inherent to latex. Elastomers, which include silicone and latex, are rubbery thermosetting compounds. As a carbon-based substance, latex can be produced by synthesizing petroleum or by naturally extracting it from rubber trees. Conversely, silicone is an inorganic polymer with an oxygen–silicon backbone. When compared with the properties of silicone illustrated in Table 2, latex has five times higher tensile strength (25 MPa) and two times higher elongation (830%).^33^

Air permeability is a crucial variable for comparing and evaluating the breathability of fabrics. It quantifies the rate of airflow through a specified area under a given air pressure difference across the material. The air permeability is influenced by various factors, including yarn characteristics, fabric construction parameters, and bulk characteristics such as thickness, mass per unit area, and porosity. An essential factor affecting the openness of the fabric structure is the gaps between the yarns.^34–36^

Upon analyzing the air permeability results, a reduction in air permeability was observed following both treatments. This decrease can be attributed to the increase in mass per unit area after coating and the presence of the coating around the fabric surface and spacer yarns. Consequently, the interstices between the two fabric surfaces decreased, creating a more restrictive pathway for air to pass through the fabric. Regardless of silicone or latex coating, the highest and lowest values of air permeability were observed in both sides of open-structured fabrics and both sides of closed fabrics, respectively. The air permeability of latex-coated fabrics was higher than that of silicone-coated ones. It is determined that the fabric surface structure affects the air permeability rather than the treatment, which explains why the air permeability values of solely both sides open-structured fabrics are very close to each other. As expected, fabrics having both sides open surfaces have higher air permeability, followed by fabrics with the face-side open and back-side closed structure and both sides closed structure (Figure 10).

**Figure 10:**
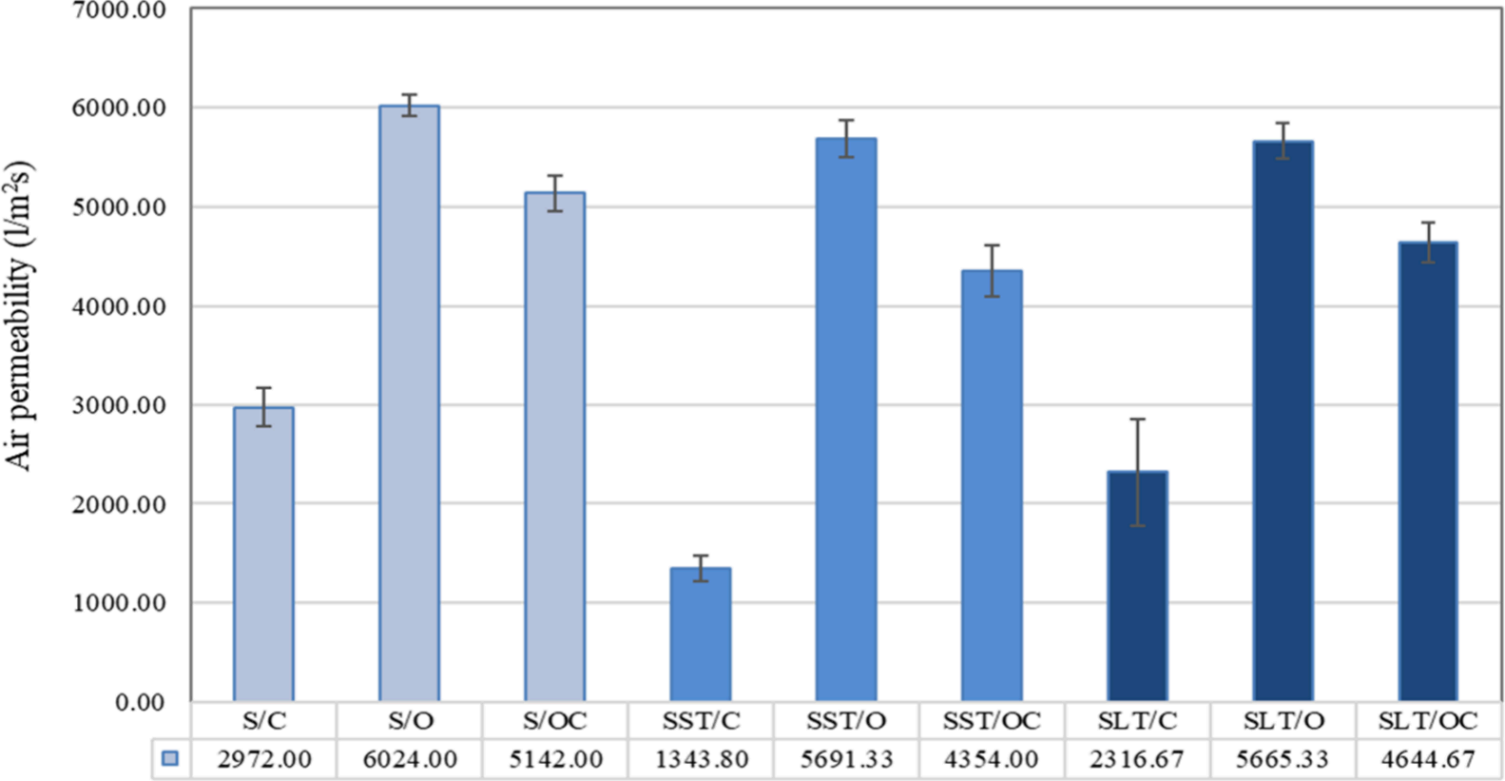
Air permeability of the samples.

Thermal resistance, which is the reciprocal of thermal conductivity (transmittance) and is used to determine the insulation value of a fabric, is described as the ratio of the heat flow per unit area normal to the faces to the temperature differential between the two surfaces of the fabric. The main factor in determining thermal insulation is the amount of trapped air. In general, more still air in the textile structure can increase the thermal resistance value of the textile and keep the body warm due to the low thermal transmission of air. However, thermal comfort is also greatly influenced by the properties of the fibers, yarns, textiles, and apparel.^37^

It was determined that the thermal resistance values of the coated fabrics were lower than those of the untreated ones. This can be explained by the amount of interstices between two fabric surfaces. Since the amount of gap in the fabric decreases after coating, the amount of trapped air also decreases. Since air has a lower thermal conductivity than the other fibers (λ_air_ = 0.026 W m^–1^ K^–1^), it is expected that structures consisting of more air will have higher thermal resistance values. In the comparison between silicone-coated and latex-coated fabrics, it was observed that the thermal resistance values of the silicone-coated fabrics were lower than those of the latex-coated ones. This difference can be attributed to the higher mass per unit area of the silicone-coated fabrics and the more substantial filling of gaps within the fabric by the silicone material. According to an assessment of the effect of surface structure on the thermal resistance value, the fabrics with both sides closed have the highest thermal resistance value, followed by fabrics with the face-side open and back-side closed and those with the both sides closed structure (Figure 11).

**Figure 11:**
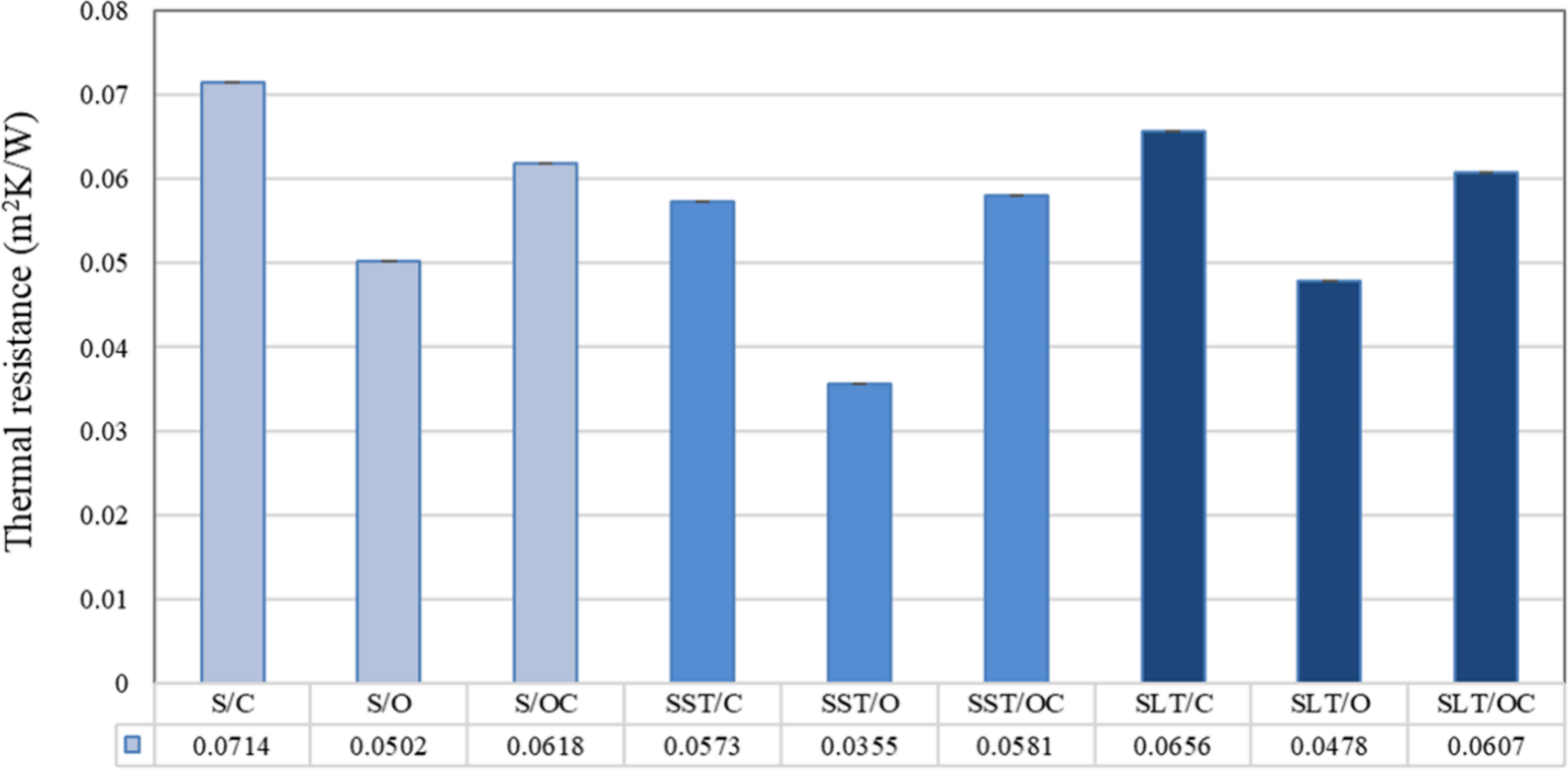
Thermal resistance of the samples.

The results of water vapor resistance and statistical evaluations of the fabrics are presented in Figure 12 and Table 3, respectively. A resistance of a material to allowing water vapor to pass through is measured by its vapor resistance. Since water vapor resistance is expressed as the resistance of fabrics to water vapor permeability, it can be concluded that fabrics with high resistance have low water vapor permeability. As stated in Figure 11, the results of water vapor resistance showed a tendency contradictory to that of air permeability. The fabric density and especially surface structure have significant effect on water vapor resistance values. The higher density and closed structure led to a significant increase in water vapor resistance. It was observed that there was an increase in the water vapor resistance of the fabrics after coating. The effect of coating on water vapor resistance was observed especially in fabrics with both sides closed surfaces, whereas this was not observed predominantly in fabrics with both sides open surfaces and fabrics with the face-side open and back-side closed structure. This situation might be explained by the mass per unit area and interstices in the fabric structure. After coating, an increase in the mass per unit area and a decrease of the interstices in the fabric structure led to an increase in the water vapor resistance of the fabrics. Statistical analysis showed that the type of coating does not have any effect on the water vapor resistance characteristic of the fabrics. Fabrics with both sides open surfaces had the lowest water vapor resistance resulting in a higher water vapor permeability, whereas fabrics with both sides closed surfaces had the highest water vapor resistance.

**Figure 12:**
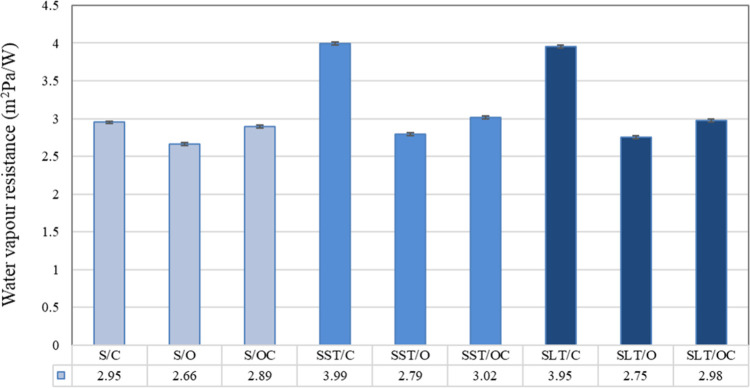
Water vapor resistance of the samples.

### 3.2 FTIR and SEM Analyses

The morphological changes of the untreated and treated fabrics are shown in Figures 13 and 14. SEM analysis revealed the uniform deposition of silicone and latex coatings on the surfaces of the treated fabrics (Figure 13). This morphological alteration indicates enhanced surface coverage, potentially improving qualities including flexibility, mechanical durability, impact absorption, and interfacial adhesion in medical protective textile applications.

**Figure 13:**
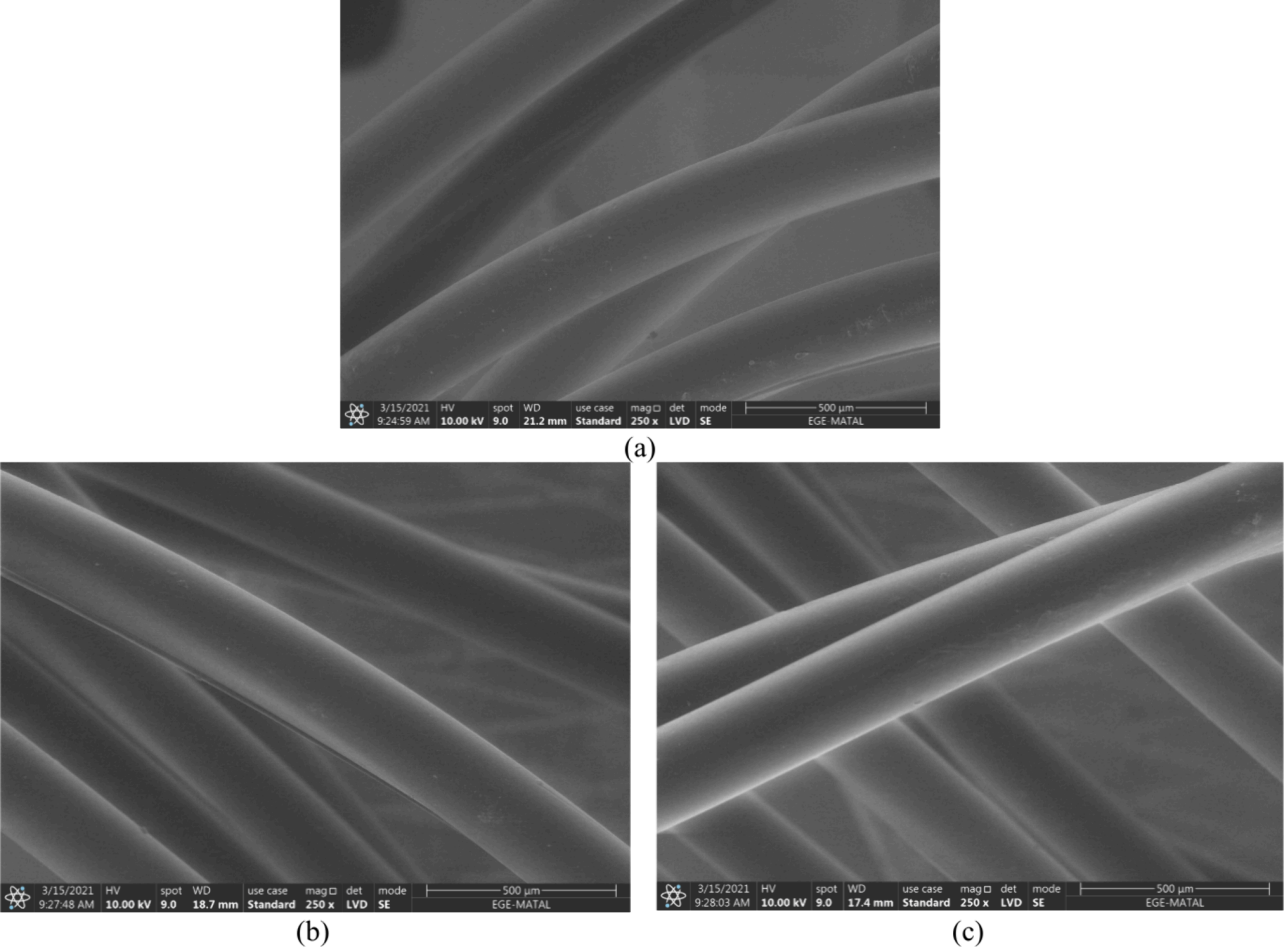
SEM images of the samples: (a) untreated, (b) treated with silicone, and (c) treated with latex.

**Figure 14:**
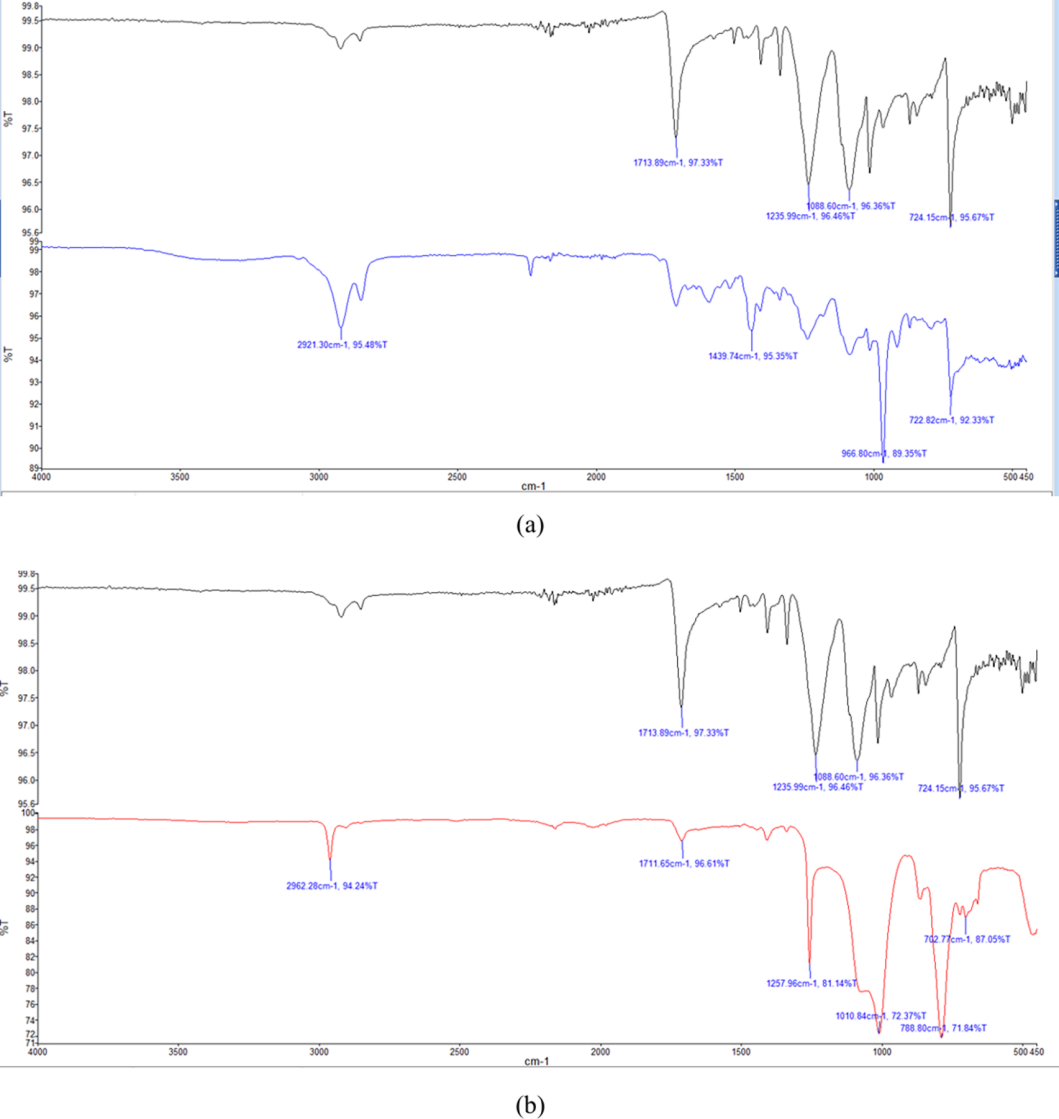
FTIR spectra of (a) silicone-treated and untreated fabrics and (b) latex-treated and untreated fabrics.

The blue spectrum (silicone-treated) features a distinct absorption band of nearly 2920 cm^–1^, corresponding to the asymmetric stretching vibration of −CH_2_/–CH_3_ groups. This is a clear indication of aliphatic methyl groups originating from the silicone coating—such as those found in polydimethylsiloxane (PDMS) chains. The typically observed asymmetric −CH_3_ stretching bands around 2950–2960 cm^–1^ in PDMS coatings were also consistent with the literature.^38,39^ The broad peak between 1080 and 1050 cm^–1^ was ascribed to the asymmetric stretching of the −Si–O–Si– vibration in the silicone-treated sample.^40^

The FTIR spectrum of the latex-treated sample (shown in red) displays the typical bands of polyisoprene. The asymmetric stretching vibrations of −CH_3_ and −CH_2_ groups, which are abundant in hydrocarbon chains in rubber matrices, induce high absorption near 2960 cm^–1^. The other prominent bands around 1010 cm^–1^ correspond to the stretching vibration of C–C, which supports the presence of unsaturated hydrocarbon backbones common in polyisoprene and related elastomers.^40,41^

The SEM images and FTIR spectra of the samples indicate the successful coating of latex and silicone on the fabric surface, as proven by morphological changes and the presence of characteristic functional groups.

## 4 Conclusions

This study reports an evaluation of the effectiveness of hip protector pads in terms of the performance properties of warp knitted spacer fabrics treated with silicone and latex, which are designed for hip protective pads. The force attenuation capacity, thermo-physiological comfort-related performance, and long-term performance were investigated. This study involved the construction of a drop weight impact tester with an upper leg profile, following the guidelines of the BS EN 1621 standard, to evaluate the force attenuation capacity of hip protective pads. The impact energy levels were varied to assess the performance of the pads on an anatomically realistic hip model, as there is currently no existing standard for determining the effectiveness of hip protectors. The findings of this study stated that treated warp knitted spacer fabrics have lower transmitted force values, resulting in a higher force attenuation capacity than untreated warp knitted spacer fabrics. The both sides open-structured, silicone-treated warp knitted spacer fabric had the lowest transmitted force values and therefore best impact protection attributes, similar to those of two different brands of hip protector supplied from the market. Since the hip protective pads were worn close to the skin and worn during the whole day, properties such as air permeability, thermal resistance, water vapor resistance for thermo-physiological comfort, compression resistance, and permanent deformation for lifetime usage assessments are very important parameters for the effectiveness of hip protective pads. In comparison to conventional hip protectors, in the fabric with the both sides open structure, the surface morphology and distance between the two fabric surfaces allows heat and vapor to be transferred easily from the skin to the environment. Due to the higher air permeability, medium compression resistance and permanent deformation, lower thermal resistance, and water vapor resistance of the both sides open-structured, silicone- or latex-treated warp knitted spacer fabrics, they are preferred for use as hip protective pads in order to provide a long life span and prevent poor compliance of users. Based on the findings of the experiments, warp knitted spacer textiles treated with either silicone or latex might be used successfully as alternatives to conventional hip protective pads.

The findings highlight the significance of comfort and durability attributes in hip protective pads intended for continuous wear by elderly or disabled patients as there have been numerous user complaints related to wearing comfort. In subsequent investigations, employing individual interviews, surveys, and clinical trials would be instrumental in comprehensively documenting the merits and drawbacks of the developed hip protectors.
